# SUBJECTIVE COGNITIVE DECLINE AND ITS IMPACTS AMONG MIDDLE-AGED AND OLDER WOMEN VETERANS AND NONVETERANS

**DOI:** 10.1093/geroni/igae098.3326

**Published:** 2024-12-31

**Authors:** Amy Henion, Amanda Cheney, Kim Sundstrom, Mohammed Alshanbari, Erin Bouldin

**Affiliations:** University of Utah, Salt Lake City, Utah, United States; University of Utah, Orem, Utah, United States; University of Utah, Salt Lake City, Utah, United States; University of Utah, Salt Lake City, Utah, United States; University of Utah, Salt Lake City, Utah, United States

## Abstract

Veterans more frequently experience traumatic brain injuries than their non-veteran peers and therefore may be at higher risk of cognitive decline. Little is known about subjective cognitive decline (SCD) among women veterans. We aimed to describe the prevalence and impacts of SCD among women veterans and compare them with non-veteran women using 2021-2022 Behavioral Risk Factor Surveillance System data from 29 jurisdictions that included the optional Cognitive Decline module. Respondents aged ≥45 years were classified as having SCD if they experienced increased memory problems or confusion over the past year. We calculated weighted estimates among 1,905 women veterans (n=268 with SCD) and 66,880 women non-veterans (n=7,609 with SCD). Women veterans were slightly younger than non-veterans (mean 61.8 versus 64.0 years, p=0.045) and more often had depression history (65.8% versus 47.9%, p=0.003). Other demographic and health characteristics – including risk factors for dementia – were similar. SCD prevalence was 13.3% among veterans and 11.7% among non-veterans (p=0.24). Among women with SCD, there were no significant differences in SCD-related social or household limitations by veteran status. Similarly, the prevalence of needing help because of SCD was similar for women veterans and non-veterans (34.6% and 35.4%, p=0.89). Veterans had more frequently discussed their SCD with a healthcare provider than non-veterans (65.7% versus 47.4%, p< 0.001). SCD prevalence and SCD-related impacts were similar among veteran and non-veteran women. The question of why veterans are more likely to discuss their SCD with providers warrants exploration and may inform strategies to facilitate such discussions in other groups.

